# Timing of hospice care initiation and aggressive care utilization in patients with cancer: a retrospective nationwide study in Korea

**DOI:** 10.3389/fpubh.2026.1909398

**Published:** 2026-08-18

**Authors:** Boram Kim, Woorim Kim, Dae Hun Ryu, Jin Young Choi

**Affiliations:** 1 National Hospice Center, National Cancer Control Institute, National Cancer Center, Goyang, Republic of Korea; 2 Department of Hospice and Palliative Service, National Cancer Center, Goyang, Republic of Korea; 3 Cancer Knowledge and Information Center, National Cancer Control Institute, National Cancer Center, Goyang, Republic of Korea

**Keywords:** aggressive care, cancer, end-of-life, hospice, palliative care

## Abstract

**Background:**

Timely hospice initiation may reduce aggressive end-of-life (EOL) care, but studies on this topic are limited. This study investigated the association between time-to-hospice use and aggressive care utilization at EOL in terminally ill patients with cancer.

**Methods:**

Using linked K-CURE claims and cancer registry data, this retrospective cohort study in South Korea included cancer patients who received hospice care between 2015 and 2022. Hospice timing was defined as the interval between the last systemic anticancer therapy (SACT), including cytotoxic chemotherapy, hormonal/endocrine therapy, targeted therapy, and immunotherapy, and the first use of hospice care. The interval was categorized as ≤ 30 days, 31–60 days, 61–90 days, and >90 days. Aggressive care outcomes included intensive care unit (ICU) admission, emergency department (ED) visits ≥2 times, and a composite measure of these outcomes occurring before hospice initiation. Multivariable logistic regression analyses were conducted, adjusting for sociodemographic and clinical variables.

**Results:**

Among 37,996 hospice users, 54.7% initiated hospice care more than 30 days after their last SACT, and this proportion exceeded 50% throughout most of the study period. The proportion initiating hospice care >90 days after the last SACT increased over time (annual percentage change, 8.2%; *p* for trend = 0.0001). ICU admission and ED visits ≥2 times before hospice initiation occurred in 3.4 and 16.4% of the patients, respectively. Compared with hospice initiation within 30 days after the last SACT, progressively higher odds of ICU admission, ED visits ≥2 times, and the composite outcome were observed with longer intervals: at 31–60 days (ORs, 3.515, 5.877, and 5.285, respectively), 61–90 days (ORs, 5.678, 13.610, and 11.806), and >90 days (ORs, 9.108, 31.127, and 25.174), indicating a clear dose-response relationship.

**Conclusions:**

Delayed hospice initiation was associated with increased use of aggressive EOL care, supporting the need for timely transition to hospice care.

## 1 Introduction

Cancer is the leading cause of mortality worldwide and places a considerable psychological burden on patients and their families (1, 2). Concurrently, population aging is likely to increase the demand for effective end-of-life (EOL) care (3). Under such circumstances, hospice care can help address the needs of EOL care by providing symptom management and caregiver support rather than focusing solely on intensive treatment (4). The need for high-quality EOL care has increased recognition of hospice services for their advantages in symptom management and patient satisfaction (5, 6). Unsurprisingly, the utilization of hospice services has increased in many aging countries such as South Korea (7). Currently, hospice care is provided by the National Health Insurance (NHI) in Korea to terminally ill individuals with cancer, acquired human immunodeficiency syndrome, chronic obstructive pulmonary disease or other chronic respiratory diseases, and chronic liver cirrhosis (8, 9). Most patients utilizing hospice services are those with cancer.

The quality of care provided at the EOL stage has been cited as an essential patient outcome (10). The provision of aggressive interventions at the EOL stage, including intensive care unit (ICU) admissions and emergency department (ED) visits, is widely recognized as an indicator of poor-quality care (11, 12). Previous studies have reported that the timing of palliative care initiation at EOL in patients with cancer may be associated with the rates of aggressive care, in which early palliative care is related to decreased ICU admissions (13). A study conducted in Canada similarly demonstrated that early palliative care was associated with lower utilization of aggressive EOL care, underscoring the importance of implementation strategies that enhance the quality of EOL care while promoting efficient use of healthcare resources (14). Specifically, this study cited ED use, hospitalization, or intensive care unit admission in the last 30 days of life as indicators of aggressive care (14). These tendencies suggest the potential importance of promoting early palliative care (15).

Aggressive EOL care can exert a substantial burden on patients, the healthcare system, and society. The use of intensive medical care has been associated with lower quality of life and worse symptom control in patients, along with poorer mental health in caregivers (16–18). Aggressive EOL care is also important in terms of the healthcare system considering that healthcare spending can increase noticeably a few months prior to death, with a substantial portion of these costs being attributable to acute medical services (19, 20). As palliative care at EOL has been reported to enhance the quality of life of patients with cancer by reducing unnecessary aggressive treatment, a need exists to explore the potential impact of the timely provision of hospice services on aggressive care utilization in patients with cancer (21).

This study aimed to investigate the association between time-to-hospice use and aggressive care utilization at EOL in terminally ill patients with stomach, colorectal, liver, pancreatic, and lung cancers using nationwide data. These cancers were included because they are the five most common causes of cancer-related deaths in Korea (22). Aggressive care was identified through ICU admissions and ED visits as they have been recognized by the Organization for Economic Cooperation and Development (OECD) as indicators of aggressive EOL care. Between 30 and 65% of people were reported to have been admitted to the ICU or visited the ED during the last 30 days of life (23). Although hospice care in Korea is generally provided in the context of foregoing cardiopulmonary resuscitation and other life-sustaining treatments, which reduces the likelihood of ICU admissions, ICU admission was included as this does not diminish its relevance as an outcome (8). As ICU admission is internationally established as a validated indicator of aggressive EOL care, it can reflect high intensity or goal discordant care despite hospice enrollment (23, 24). We hypothesized that patients who used hospice services at an earlier stage would show lower rates of aggressive care utilization.

## 2 Materials and methods

### 2.1 Data sources

This retrospective population-based cohort study used the Cancer Public Library Database (CPLD), established by the Korean Clinical Data Utilization for Research Excellence (K-CURE) project. The K-CURE CPLD links four nationwide data sources: the Korea National Cancer Incidence Database from the Korea Central Cancer Registry (KCCR), cause-of-death data from Statistics Korea, the National Health Information Database from the National Health Insurance Service (NHIS), and claims data from the Health Insurance Review and Assessment Service (HIRA) (25). These datasets were linked using encrypted resident registration numbers. They contained information on cancer registration, age at diagnosis, sex, cancer type, SEER summary stage, morphology code, treatment modalities, cause and date of death, results of general health examinations, and health insurance eligibility status. This study was approved by the Institutional Review Board of the National Cancer Center (NCC2025-0056).

### 2.2 Study population

We first identified patients with cancer-related claims between January 1, 2015, and December 31, 2022. Cancer-related claims were defined using ICD-10 codes (C00–C97) to construct the source population. The final analytic cohort was subsequently restricted to five major high-mortality cancer types: stomach, colorectal, liver, pancreatic, and lung cancers. The starting date of January 1, 2015, was used to define the overall observation period for identifying cancer-related claims, eligibility information, and systemic anticancer therapy (SACT)-related records. Hospice care use was ascertained using hospice-specific reimbursement codes, which were captured after the implementation of National Health Insurance reimbursement for hospice care in July 2015.

Hospice care users were identified by hospice-related service codes (Supplementary Table S1). For each patient, we derived the date of first hospice care use and hospice care type. Because hospice care in Korea includes inpatient, home-based, consultation-based, and mixed service patterns, we used the term first hospice care (26–28).

To preserve the temporal sequence between SACT and hospice care, cancer claims after the first hospice care use date were not used to define prehospice cancer-related information. Patients were included in the final analytic cohort if they satisfied the following criteria: use of hospice care, a cancer-related special registration code (V193, V194), coverage by the National Health Insurance or Medical Aid, and a SACT-related record before hospice care use that matched the cancer type recorded at the last cancer-related medical utilization before hospice care use. SACT was identified using the claims data and included cytotoxic chemotherapy, hormonal/endocrine therapy, targeted therapy, and immunotherapy. The detailed operational definitions and reimbursement codes are provided in Supplementary Table S1. The analysis code identified the most recent treatment-related date before hospice care use and classified patients as having a matched treatment record when the cancer type in the SACT-related claim matched the last cancer diagnosis type. Patients with implausible temporal ordering, including those whose last SACT date was after their last cancer-related medical utilization date, were excluded.

**Figure 1:**
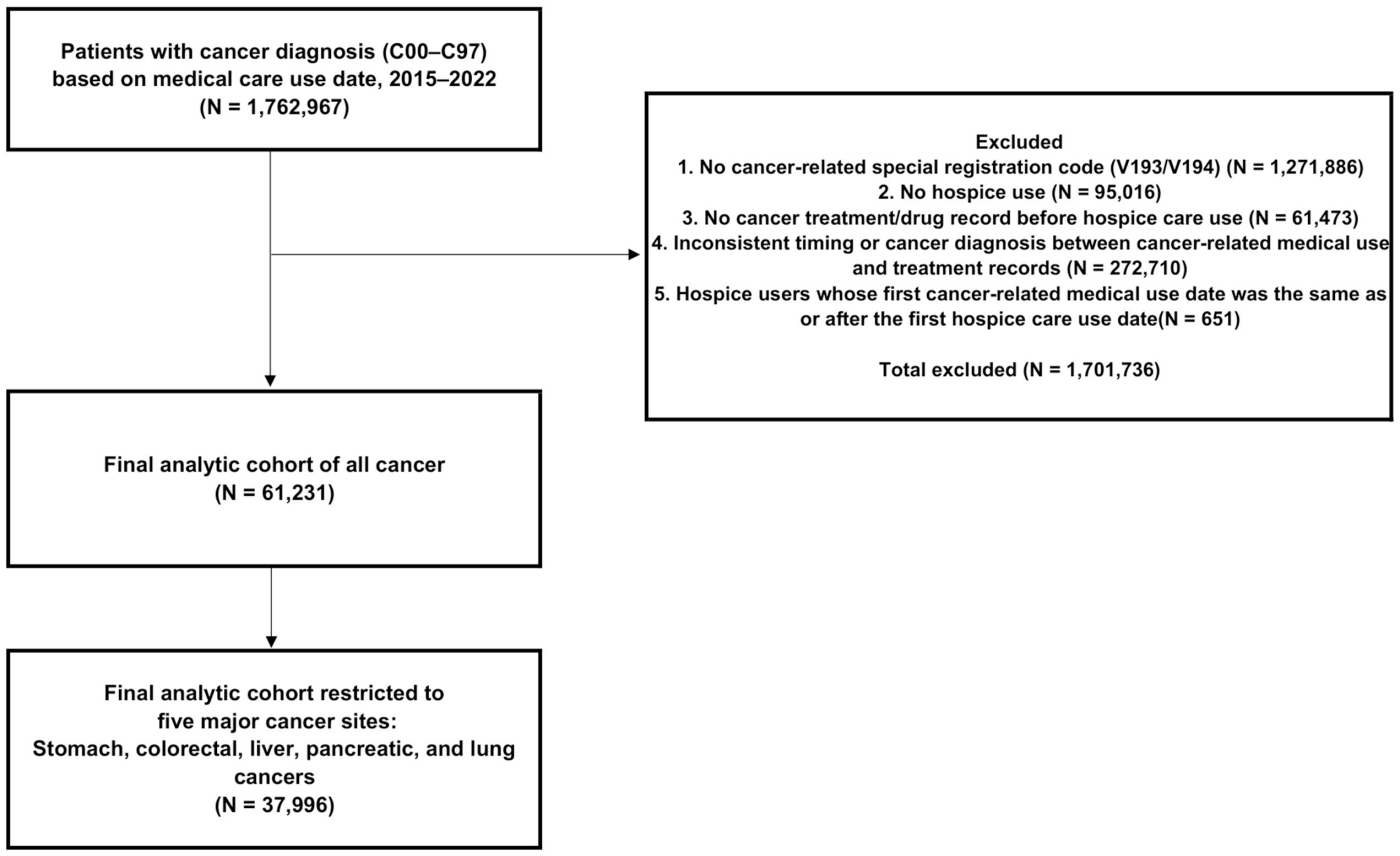
Flow chart of the selection process for the final analytic cohort.

The final analysis was restricted to five major high-mortality cancer types in Korea: stomach, colorectal, liver, pancreatic, and lung cancers. The ICD-10 codes used were as follows: C16 for stomach cancer; C18–C20 for colorectal cancer; C22 for liver cancer; C25 for pancreatic cancer; and C33–C34 for lung cancer (22). These cancer types were selected because they represent clinically important high-mortality solid tumors in Korea. According to national projections for 2022, lung, liver, colorectal, pancreatic, and stomach cancers are among the leading causes of cancer-related death in men, while lung, colorectal, pancreatic, breast, liver, and stomach cancers are the major causes of cancer-related death in women. The detailed participant selection process is presented in Figure 1.

### 2.3 Variables

The primary exposure was the timing of hospice care use, defined as the interval between the last SACT date before hospice care and the first hospice care date. In the analyses, hospice timing was categorized as ≤ 30 days, 31–60 days, 61–90 days, and >90 days, with ≤ 30 days used as the reference category. The 30-day interval was selected as a clinically interpretable period based on prior EOL oncology studies that commonly evaluated the use and intensity of aggressive care during the last month of life (12, 29, 30). Three binary outcomes were evaluated: ICU admission, ED visits ≥2 times, and the composite outcome of ICU admission or ED visits ≥2 times. These outcomes were identified during the interval between the last SACT date before hospice care and the first hospice care use date. The reimbursement codes used to define these variables are presented in Supplementary Table S1.

These outcomes were selected because ED visits and ICU admissions near EOL are widely used claims-based indicators of high-intensity care and potentially suboptimal transition to palliative or hospice care (12, 29, 31, 32). Covariates were selected *a priori* based on clinical relevance and previous studies evaluating hospice or palliative care timing and aggressive EOL care, in which demographic characteristics, socioeconomic or geographic factors, cancer type, disease stage, comorbidity, care setting, and calendar period were commonly considered potential confounders (10, 14, 29). The sociodemographic variables included sex, age, insurance type, and area of residence. Age was categorized as < 50, 50–59, 60–69, and ≥70. The insurance type was categorized as employee insurance, self-employed insurance, or Medical Aid. Residential areas were categorized as capital cities, metropolitan areas, or rural areas. The type of medical institution at the last SACT before hospice care was also included and categorized as tertiary general hospital, general hospital, or hospital/clinic/other. Hospice care types were categorized as inpatient, home-based, consultation-based, or mixed. Hospice entry year was defined as the calendar year of the first hospice care use. Cancer-related variables included the primary cancer site and SEER stage at primary diagnosis. The primary cancer sites were categorized as stomach, colorectal, liver, pancreas, or lung. The SEER stage was categorized as *in situ*, localized, regional, distant, or unknown. The comorbidity burden was assessed using the modified Charlson Comorbidity Index (CCI). The modified CCI was calculated from the diagnosis codes recorded 365 days before the last SACT date, excluding the index date itself (33, 34).

### 2.4 Statistical analysis

Baseline characteristics were summarized according to hospice timing. Categorical variables are presented as frequencies and column percentages. Differences across hospice timing groups were assessed using a chi-square test. The annual distribution of hospice timing was summarized using four descriptive timing categories: ≤ 30 days, 31–60 days, 61–90 days, and >90 days. For each category, *p* for trend and Annual Percentage Change (APC) were estimated (exp(β)−1) × 100, where β was the coefficient for calendar year from a log-linear regression model of the annual proportion (35). These additional categories were used to describe the clinically interpretable gradients of the SACT-to-hospice transition interval. For each calendar year of the first hospice care use, the number and column percentages of patients in each timing category were reported. Multivariate logistic regression models were used to evaluate the association between hospice timing and prior use of aggressive care. Three separate models were fitted for ICU admissions, ED visits ≥2 times, and the composite outcome of ICU admissions or ED visits ≥2 times. Each model was adjusted for sex, age group, insurance type, residential area, hospice care type, hospice entry year, type of medical institution, primary cancer site, SEER stage at primary diagnosis, and the modified CCI group. Unadjusted logistic regression models were also fitted to evaluate the crude association between hospice timing and each aggressive care outcome, with hospice timing as the only independent variable. The results were reported as odds ratios (ORs) and 95% confidence intervals (CIs). All analyses were performed using SAS version 9.4 (SAS Institute Inc., Cary, NC, USA) and R software version 4.5.1.

## 3 Results

### 3.1 Baseline characteristics

A total of 37,996 hospice users with five major cancers were included in the final cohort. Table 1 presents the baseline characteristics of the study population by the four hospice timing categories. Among the 37,996 hospice users, 17,222 (45.3%) used hospice care within 30 days of the last SACT, whereas 20,774 (54.7%) used hospice care after more than 30 days. Overall, 63.3% of the patients were men, 48.0% were aged ≥70 years, and distant-stage disease was the most common SEER stage at the primary diagnosis (48.6%). Lung cancer was the most common primary cancer site (27.5%). The modified CCI scores were 0 in 30.0%, 1 in 37.5%, and ≥2 in 32.6%. Patients in the ≤ 30 days group were older and had a higher proportion of distant-stage disease and a modified CCI score ≥ 2 than those with longer hospice timing intervals.

**Table 1:** Sociodemographic and clinical characteristics of hospice users in cancer patients.

Variable	Total	≤ 30 days	31–60 days	61–90 days	>90 days	*P*-value
	(*N* = 37,996)	(*N* = 17,222)	(*N* = 7,216)	(*N* = 3,211)	(*N* = 10,347)	
Age, *n* (%)						< 0.0001
< 50	2,520 (6.6)	739 (4.3)	422 (5.8)	254 (7.9)	1,105 (10.7)	
50–59	6,296 (16.6)	2,342 (13.6)	1,204 (16.7)	577 (18.0)	2,173 (21.0)	
60–69	10,929 (28.8)	4,768 (27.7)	2,234 (31.0)	938 (29.2)	2,989 (28.9)	
≥70	18,251 (48.0)	9,373 (54.4)	3,356 (46.5)	1,442 (44.9)	4,080 (39.4)	
Sex, *n* (%)						< 0.0001
Male	24,068 (63.3)	11,215 (65.1)	4,553 (63.1)	1,954 (60.9)	6,346 (61.3)	
Female	13,928 (36.7)	6,007 (34.9)	2,663 (36.9)	1,257 (39.1)	4,001 (38.7)	
Insurance type, *n* (%)						< 0.0001
Self-employed insured	23,237 (61.2)	10,705 (62.2)	4,414 (61.2)	1,926 (60.0)	6,192 (59.8)	
Employee insured	11,637 (30.6)	5,168 (30.0)	2,244 (31.1)	1,009 (31.4)	3,216 (31.1)	
Medical aid	3,122 (8.2)	1,349 (7.8)	558 (7.7)	276 (8.6)	939 (9.1)	
Hospice care type, *n* (%)						< 0.0001
Inpatient	27,416 (72.2)	12,618 (73.3)	5,360 (74.3)	2,364 (73.6)	7,074 (68.4)	
Home-based	3,458 (9.1)	1,530 (8.9)	627 (8.7)	277 (8.6)	1,024 (9.9)	
Consultation-based	968 (2.5)	438 (2.5)	168 (2.3)	85 (2.6)	277 (2.7)	
Mixed	6,154 (16.2)	2,636 (15.3)	1,061 (14.7)	485 (15.1)	1,972 (19.1)	
Type of medical institution, *n* (%)						< 0.0001
Tertiary general hospital	12,112 (31.9)	5,300 (30.8)	2,244 (31.1)	995 (31.0)	3,573 (34.5)	
General hospital	15,829 (41.7)	7,347 (42.7)	3,036 (42.1)	1,305 (40.6)	4,141 (40.0)	
Hospital/clinic/other	10,055 (26.5)	4,575 (26.6)	1,936 (26.8)	911 (28.4)	2,633 (25.4)	
Residential area, *n* (%)						< 0.0001
Capital city area	6,176 (16.3)	2,573 (14.9)	1,252 (17.4)	552 (17.2)	1,799 (17.4)	
Metropolitan area	11,733 (30.9)	5,415 (31.4)	2,212 (30.7)	956 (29.8)	3,150 (30.4)	
Rural area	20,087 (52.9)	9,234 (53.6)	3,752 (52.0)	1,703 (53.0)	5,398 (52.2)	
Primary cancer site, *n* (%)						< 0.0001
Stomach	7,186 (18.9)	2,699 (15.7)	1,267 (17.6)	600 (18.7)	2,620 (25.3)	
Colon and rectum	7,429 (19.6)	2,834 (16.5)	1,331 (18.4)	647 (20.1)	2,617 (25.3)	
Liver	6,622 (17.4)	3,327 (19.3)	1,314 (18.2)	583 (18.2)	1,398 (13.5)	
Pancreas	6,317 (16.6)	3,203 (18.6)	1,342 (18.6)	530 (16.5)	1,242 (12.0)	
Lung	10,442 (27.5)	5,159 (30.0)	1,962 (27.2)	851 (26.5)	2,470 (23.9)	
SEER stage at primary diagnosis, *n* (%)						< 0.0001
Localized	4,582 (12.1)	2,096 (12.2)	841 (11.7)	399 (12.4)	1,246 (12.0)	
*In situ*	92 (0.2)	41 (0.2)	11 (0.2)	12 (0.4)	28 (0.3)	
Regional	11,812 (31.1)	4,950 (28.7)	2,102 (29.1)	996 (31.0)	3,764 (36.4)	
Distant	18,450 (48.6)	8,680 (50.4)	3,644 (50.5)	1,551 (48.3)	4,575 (44.2)	
Unknown	3,060 (8.1)	1,455 (8.4)	618 (8.6)	253 (7.9)	734 (7.1)	
Charlson comorbidity index score, *n* (%)						< 0.0001
0	11,389 (30.0)	4,818 (28.0)	2,135 (29.6)	988 (30.8)	3,448 (33.3)	
1	14,231 (37.5)	6,374 (37.0)	2,699 (37.4)	1,236 (38.5)	3,922 (37.9)	
≥2	12,376 (32.6)	6,030 (35.0)	2,382 (33.0)	987 (30.7)	2,977 (28.8)	
	(*N* = 37,996)	(*N* = 17,222)	(*N* = 7,216)	(*N* = 3,211)	(*N* = 10,347)	
Hospice entry year, *n* (%)						< 0.0001
2015	1,331 (3.5)	386 (2.2)	231 (3.2)	157 (4.9)	557 (5.4)	
2016	4,157 (10.9)	2,008 (11.7)	797 (11.0)	340 (10.6)	1,012 (9.8)	
2017	5,445 (14.3)	2,731 (15.9)	1,103 (15.3)	436 (13.6)	1,175 (11.4)	
2018	5,975 (15.7)	2,870 (16.7)	1,222 (16.9)	498 (15.5)	1,385 (13.4)	
2019	6,508 (17.1)	3,028 (17.6)	1,269 (17.6)	558 (17.4)	1,653 (16.0)	
2020	6,201 (16.3)	2,985 (17.3)	1,140 (15.8)	493 (15.4)	1,583 (15.3)	
2021	5,014 (13.2)	2,056 (11.9)	897 (12.4)	447 (13.9)	1,614 (15.6)	
2022	3,365 (8.9)	1,158 (6.7)	557 (7.7)	282 (8.8)	1,368 (13.2)	
ICU admission, *n* (%)						< 0.0001
No	36,706 (96.6)	17,071 (99.1)	6,997 (97.0)	3,057 (95.2)	9,581 (92.6)	
Yes	1,290 (3.4)	151 (0.9)	219 (3.0)	154 (4.8)	766 (7.4)	
ED visits ≥2 times, *n* (%)						< 0.0001
No	31,754 (83.6)	16,852 (97.9)	6,364 (88.2)	2,455 (76.5)	6,083 (58.8)	
Yes	6,242 (16.4)	370 (2.1)	852 (11.8)	756 (23.5)	4,264 (41.2)	

Values are presented as *n* (%). 30 days was defined as the interval from the last SACT date before hospice use to the first hospice care use date; SEER, Surveillance, Epidemiology, and End Results.

### 3.2 Annual distribution of hospice care timing

Table 2 presents the annual distribution of the interval between the last SACT date and the first use of hospice care. Overall, 17,222 patients (45.3%) initiated hospice care within 30 days of their last SACT, followed by >90 days for 10,347 patients (27.2%), 31–60 days for 7,216 patients (19.0%), and 61–90 days for 3,211 patients (8.5%). The ≤ 30 days group decreased over time (APC, −4.3% per year; *p* for trend < 0.0001), as did the 31–60 days group (APC, −2.5% per year; *p* for trend = 0.0002). The 61–90 days group showed no significant temporal trend (APC: −0.7% per year; *p* for trend = 0.4408). In contrast, the >90 days group showed a marked upward trend over time (APC: 8.2% per year; *p* for trend = 0.0001), with the proportion increasing to 40.7% by 2022. The 2015 distribution should be interpreted cautiously because National Health Insurance coverage for hospice care costs began in July 2015.

**Table 2:** Annual distribution of interval from last Systemic Anticancer Therapy to hospice care initiation.

Timing of hospice initiation	Total *n* (%)	2015 *n* (%)	2016 *n* (%)	2017 *n* (%)	2018 *n* (%)	2019 *n* (%)	2020 *n* (%)	2021 *n* (%)	2022 *n* (%)	*p* for trend	APC per year
Total	37,996 (100.0)	1,331 (100.0)	4,157 (100.0)	5,445 (100.0)	5,975 (100.0)	6,508 (100.0)	6,201 (100.0)	5,014 (100.0)	3,365 (100.0)		
≤ 30 days	17,222 (45.3)	386 (29.0)	2,008 (48.3)	2,731 (50.2)	2,870 (48.0)	3,028 (46.5)	2,985 (48.1)	2,056 (41.0)	1,158 (34.4)	< 0.0001	−4.30%
31–60 days	7,216 (19.0)	231 (17.4)	797 (19.2)	1,103 (20.3)	1,222 (20.5)	1,269 (19.5)	1,140 (18.4)	897 (17.9)	557 (16.6)	0.0002	−2.50%
61–90 days	3,211 (-8.5)	157 (11.8)	340 (8.2)	436 (8.0)	498 (8.3)	558 (8.6)	493 (8.0)	447 (8.9)	282 (8.4)	0.4408	−0.70%
>90 days	10,347 (27.2)	557 (41.8)	1,012 (24.3)	1,175 (21.6)	1,385 (23.2)	1,653 (25.4)	1,583 (25.5)	1,614 (32.2)	1,368 (40.7)	0.0001	8.20%

Data are presented as *n* (%). Timing of hospice care initiation was calculated as the number of days from the last SACT date before hospice use to the first hospice care use date; APC, annual percentage change.

### 3.3 Association between timing of hospice care and aggressive care use

Table 3 presents the results of multivariate logistic regression models evaluating the association between hospice care initiation and the use of aggressive care before hospice care. During the interval between the last SACT date and the first hospice care use, 1,290 patients (3.4%) were admitted to the ICU, and 6,242 patients (16.4%) had two or more ED visits. A composite outcome of ICU admission or ED visits ≥2 times was observed in 6,966 patients. After adjusting for sex, age group, insurance type, residential area, hospice care type, hospice entry year, type of medical institution, primary cancer site, SEER stage at primary diagnosis, and modified CCI group, longer intervals from the last SACT to hospice initiation were associated with progressively higher odds of aggressive care use before hospice care. Compared to hospice initiation within 30 days, the odds of ICU admission increased for hospice initiation at 31–60 days (OR, 3.515; 95% CI, 2.850–4.335), 61–90 days (OR, 5.678; 95% CI, 4.517–7.138), and >90 days (OR, 9.108; 95% CI, 7.608–10.903). A similar gradient was observed for ED visits ≥2 times, with ORs of 5.877 (95% CI, 5.181–6.665), 13.610 (95% CI, 11.922–15.538), and 31.127 (95% CI, 27.821–34.826), respectively. For the composite outcome of ICU admission or ED visits ≥2 times, the corresponding ORs were 5.285 (95% CI, 4.732–5.902), 11.806 (95% CI, 10.486–13.291), and 25.174 (95% CI, 22.817–27.774), respectively. All associations with hospice timing were statistically significant (*p* < 0.0001).

**Table 3:** Multivariable logistic regression analysis of aggressive care according to timing of hospice initiation.

Variable	Level	ICU admission (*N* = 1,290) adjusted OR (95% CI)	*P*-value	ED visits ≥2 times, (*N* = 6,242) adjusted OR (95% CI)	*P*-value	ICU admission or ED visits≥2 times, (*N* = 6,966) adjusted OR (95% CI)	*P*-value
Timing of hospice initiation	≤ 30 days	Ref.		Ref.		Ref.	
	31–60 days	3.515 (2.850–4.335)	< 0.0001	5.877 (5.181–6.665)	< 0.0001	5.285 (4.732–5.902)	< 0.0001
	61–90 days	5.678 (4.517–7.138)	< 0.0001	13.610 (11.922–15.538)	< 0.0001	11.806 (10.486–13.291)	< 0.0001
	>90 days	9.108 (7.608–10.903)	< 0.0001	31.127 (27.821–34.826)	< 0.0001	25.174 (22.817–27.774)	< 0.0001
Age, years	< 50	Ref.		Ref.		Ref.	
	50–59	0.891 (0.708–1.121)	0.324	0.754 (0.670–0.848)	< 0.0001	0.766 (0.682–0.859)	< 0.0001
	60–69	0.952 (0.766–1.184)	0.6605	0.611 (0.545–0.684)	< 0.0001	0.642 (0.575–0.718)	< 0.0001
	≥70	0.763 (0.614–0.948)	0.0147	0.447 (0.399–0.501)	< 0.0001	0.478 (0.428–0.533)	< 0.0001
Sex	Male	Ref.		Ref.		Ref.	
	Female	0.862 (0.764–0.973)	0.016	1.040 (0.976–1.109)	0.2298	1.014 (0.954–1.078)	0.6547
Insurance type	Self-employed insured	Ref.		Ref.		Ref.	
	Employee insured	1.009 (0.890–1.143)	0.8907	1.040 (0.972–1.114)	0.2548	1.046 (0.980–1.117)	0.1731
	Medical Aid	0.862 (0.689–1.078)	0.1932	0.917 (0.814–1.033)	0.1545	0.916 (0.817–1.027)	0.1329
Hospice care type	Inpatient	Ref.		Ref.		Ref.	
	Home-based	0.459 (0.281–0.749)	0.0018	0.723 (0.590–0.887)	0.0019	0.681 (0.559–0.830)	0.0001
	Consultation-based	0.926 (0.746–1.150)	0.488	0.537 (0.474–0.608)	< 0.0001	0.556 (0.494–0.626)	< 0.0001
	Mixed	0.705 (0.589–0.844)	< 0.0001	0.667 (0.609–0.732)	< 0.0001	0.647 (0.592–0.707)	< 0.0001
Type of medical institution	Tertiary general hospital	Ref.		Ref.		Ref.	
	General hospital	1.151 (0.993–1.333)	0.0613	0.831 (0.769–0.899)	< 0.0001	0.873 (0.810–0.941)	0.0004
	Hospital/clinic/other	1.042 (0.879–1.237)	0.6344	0.818 (0.747–0.895)	< 0.0001	0.848 (0.777–0.925)	0.0002
Residential area	Capital city area	Ref.		Ref.		Ref.	
	Metropolitan area	0.833 (0.701–0.989)	0.0374	0.855 (0.779–0.939)	0.0011	0.860 (0.786–0.940)	0.0009
	Rural area	0.928 (0.795–1.082)	0.3396	0.959 (0.880–1.044)	0.3358	0.958 (0.882–1.039)	0.2995
Primary cancer site	Stomach	Ref.		Ref.		Ref.	
	Colon and rectum	1.396 (1.170–1.665)	0.0002	0.844 (0.769–0.926)	0.0003	0.891 (0.814–0.974)	0.0112
	Liver	1.382 (1.144–1.668)	0.0008	1.044 (0.942–1.156)	0.4122	1.059 (0.960–1.168)	0.2528
	Pancreas	1.159 (0.946–1.421)	0.1548	1.119 (1.010–1.239)	0.0316	1.121 (1.016–1.237)	0.0232
	Lung	1.221 (1.022–1.457)	0.0276	0.775 (0.707–0.850)	< 0.0001	0.816 (0.747–0.892)	< 0.0001
SEER stage at primary diagnosis	Localized	Ref.		Ref.		Ref.	
	*In situ*	0.476 (0.116–1.985)	0.3104	1.160 (0.632–2.131)	0.6319	0.944 (0.518–1.720)	0.8517
	Regional	0.891 (0.748–1.062)	0.1989	1.178 (1.059–1.311)	0.0027	1.135 (1.026–1.256)	0.0143
	Distant	0.628 (0.525–0.751)	< 0.0001	1.127 (1.014–1.253)	0.0266	1.038 (0.939–1.148)	0.4662
	Unknown	0.960 (0.757–1.217)	0.7354	1.080 (0.934–1.250)	0.2987	1.083 (0.944–1.243)	0.255
Charlson comorbidity index score	0	Ref.		Ref.		Ref.	
	1	1.083 (0.941–1.246)	0.2681	1.076 (0.998–1.159)	0.0526	1.090 (1.014–1.172)	0.0189
	≥2	1.175 (1.012–1.365)	0.0342	1.206 (1.112–1.308)	< 0.0001	1.204 (1.113–1.301)	< 0.0001
hospice entry year	2015	Ref.		Ref.		Ref.	
	2016	1.016 (0.736–1.403)	0.9224	0.939 (0.788–1.118)	0.4799	0.951 (0.804–1.125)	0.5583
	2017	1.019 (0.744–1.395)	0.907	1.146 (0.968–1.356)	0.1126	1.132 (0.963–1.332)	0.1328
	2018	1.085 (0.796–1.477)	0.6059	1.273 (1.079–1.504)	0.0043	1.250 (1.065–1.467)	0.0063
	2019	1.126 (0.829–1.528)	0.4482	1.353 (1.148–1.595)	0.0003	1.337 (1.141–1.567)	0.0003
	2020	1.298 (0.957–1.760)	0.0938	1.381 (1.170–1.631)	0.0001	1.399 (1.192–1.641)	< 0.0001
	2021	1.097 (0.802–1.501)	0.5623	1.217 (1.028–1.441)	0.0226	1.234 (1.049–1.452)	0.0113
	2022	0.980 (0.706–1.359)	0.902	1.524 (1.281–1.813)	< 0.0001	1.441 (1.218–1.704)	< 0.0001

ORs and 95% CIs were estimated using multivariable logistic regression. Separate models were fitted for ICU admission, ED visits ≥2 times, and the composite outcome of ICU admission or ED visits ≥2 times. Aggressive care use was assessed during the interval between the last SACT date before hospice use and the first hospice care use date.

**Figure 2:**
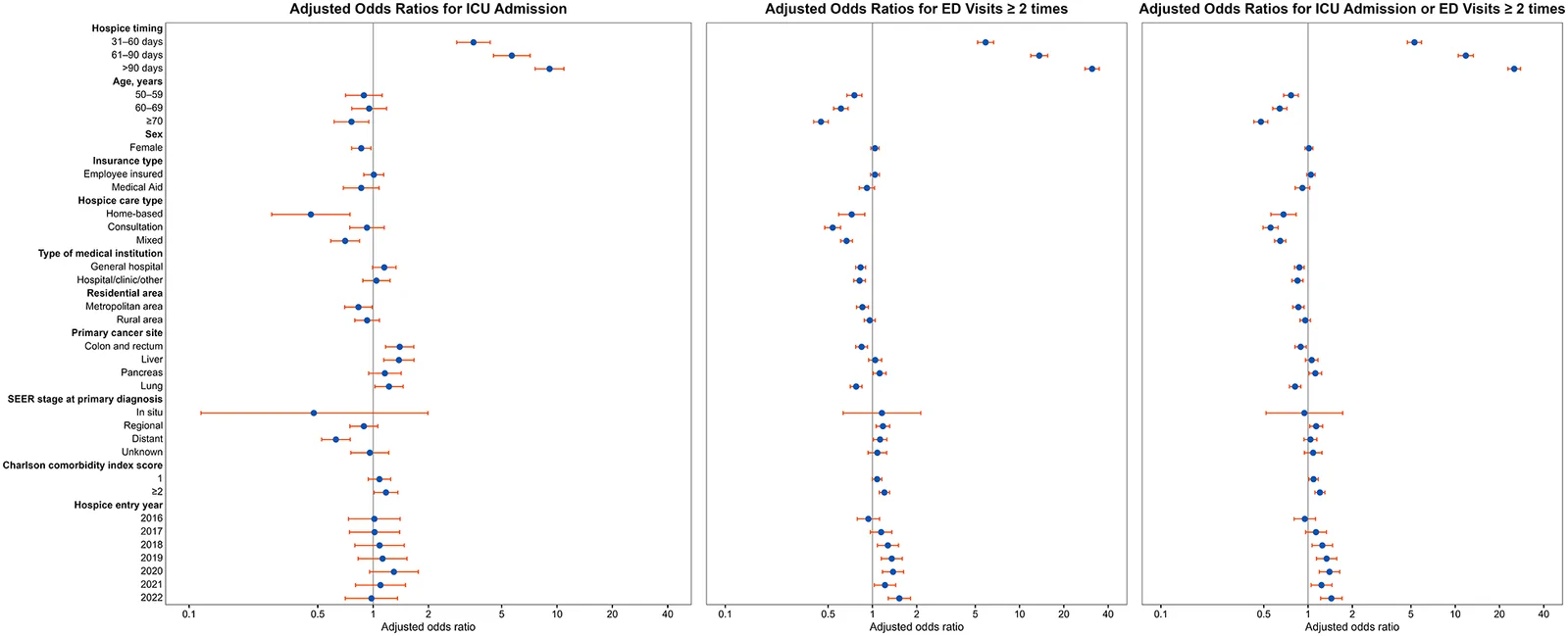
Forest plots of multivariable logistic regression analysis of aggressive care according to timing of hospice initiation.

Among the covariates included for adjustment, older age, hospice care type, primary cancer site, type of medical institution at the last SACT, and modified CCI score showed associations with one or more aggressive care outcomes. Detailed estimates for all covariates are presented in Table 3. The corresponding forest plots are shown in Figure 2.

In unadjusted analyses, longer intervals from the last SACT to hospice initiation were also associated with progressively higher odds of ICU admission, ED visits ≥2 times, and the composite outcome. These results are presented in Supplementary Table S1.

## 4 Discussion

According to this study, a substantial proportion of cancer patients initiated hospice care >30 days after their last SACT. During the study period, this proportion remained above 50% in most years, and the proportion exceeding 90 days showed an upward trend. Furthermore, a significant association was observed between hospice initiation time and aggressive care. These findings provide important insights into hospice initiation patterns among cancer patients and extend existing research by offering robust nationwide evidence. This study builds on previous studies that were confined to regional data or specific cancer types (10, 14). Moreover, using nationwide insurance claims data, this study evaluated the association between aggressive care and the different types of hospice care currently covered by the Korean National Health Insurance System. To our knowledge, this is the first study in Korea to comprehensively examine the transition from SACT to hospice initiation, including annual trends and association with aggressive EOL care.

Prior studies have primarily focused on hospice length of stay, whereas evidence regarding the interval between the last SACT and hospice initiation remains limited (7, 36, 37). Consistent with our findings, a previous Korean study reported a median interval of 75 days between the last SACT and hospice admission in patients with terminal cancer (36). Hospital use in Korea has gradually increased over time. However, the duration of hospice enrollment remains suboptimal, potentially reflecting delayed initiation of hospice care (37–39). The length of hospital stay has remained under 30 days in Korea in recent years (7). A recent international systematic review highlighted that late hospice enrollment remains prevalent across diverse healthcare settings (40). Previous studies have suggested that late hospice initiation is multifactorial and may be associated with prognostic uncertainty, continued preference for disease-directed treatment, misconceptions about hospice care, delayed EOL discussions, and healthcare system barriers (38, 41). In Korea, the cultural reluctance to discuss death, family-centered decision-making, and limited accessibility to hospice care may contribute to delayed hospice initiation (41). The increased proportion of patients initiating hospice care >90 days after their last SACT in 2021 and 2022 may partly reflect the reduced accessibility to hospice services during the COVID-19 pandemic. Previous studies have reported temporary hospice unit closures, disruptions in service continuity due to infection control policies, and a decline in overall hospice utilization during the pandemic (42). Given the multifactorial nature of delayed hospice initiation, further research on patient, physician, and healthcare system factors is needed to develop strategies for timely hospice utilization among patients with terminal cancer.

Aggressive EOL care, such as ICU admission and ED visits ≥2 times, among terminal cancer patients remains common worldwide (40) and in Korea (43). Specifically, the 16.4% prevalence of ≥2 ED visits observed in the present study was broadly comparable to the 12–14% reported in these studies. A previous nationwide Korean study further demonstrated that ED visits were not uncommon among terminal cancer patients following the last SACT (44), consistent with the findings of the present study. These findings are clinically important because aggressive EOL care is generally considered a marker of poor-quality EOL care and may impose substantial burdens on patients and healthcare systems without a meaningful survival benefit (45, 46). In Korea, aggressive EOL care remains prevalent among patients with terminal cancer, despite the enactment of the Act on Decisions on Life-Sustaining Treatment in 2018 (47), which aimed to promote hospice care and reduce futile aggressive EOL care. Collectively, these findings highlight the need for strategies to identify patients at high risk of aggressive EOL care and facilitate timely hospice initiation to improve the quality of EOL care. Nevertheless, the ICU admission rate observed in the present study (3.4%) was substantially lower than the rates reported in prior Korean and international studies (10–15%) (40, 43, 44). This difference may partly reflect our restriction to hospice users. Patients who ultimately transition to hospice care may have been more likely to have goals of care that favored less aggressive treatment. Although information on hospice referral or consultation was not available in our data, such interventions may have contributed to lower ICU admission rate. A previous study has demonstrated that earlier palliative care consultation is independently associated with lower rates of aggressive EOL care (39).

A graded association was observed between the interval from the last SACT to hospice initiation and the use of aggressive EOL care, with progressively higher odds of ICU admission, ED visits, and composite outcomes across the interval groups. These findings are broadly consistent with those of previous studies showing that palliative care initiation earlier than 3 months before death was associated with lower rates of aggressive EOL care (10, 14). Similarly, a recent Korean study reported that earlier palliative care consultation (>90 days before death) was associated with significantly lower rates of ICU admission and ED visit than late referral ( ≤ 30 days before death), consistent with our findings (48). However, unlike previous studies that evaluated the timing of hospice or palliative care in relation to death (37), our study extends this perspective by focusing on the transition period between the last SACT and hospice initiation. This transition may be particularly relevant in the Korean healthcare setting. The Korean government has promoted hospice care as a national policy since the early 2000's, including the official designation of hospice palliative care units eligible for government subsidies in 2008 (9). Furthermore, hospice care has been integrated into the universal National Health Insurance system since reimbursement for inpatient hospice care was introduced in 2015, with coverage subsequently expanded to home-based and consultation-based hospice services in Korea (7, 9, 43). Despite this development, hospice care in Korea continues to be predominantly delivered through inpatient hospice units and has largely been provided as a post-treatment service in the context of imminent EOL care, rather than being integrated concurrently from the time of serious illness diagnosis (9, 49). Therefore, the interval between the last SACT and hospice initiation may serve as a meaningful indicator of the timeliness of hospice transition, particularly in healthcare systems where hospice and palliative care remain largely focused on EOL care. Timely hospice initiation may facilitate goal-of-care discussions, advance care planning, and symptom management, thereby reducing unnecessary utilization of acute healthcare near the EOL (10).

Furthermore, home-based, consultation-based, and mixed hospice care were associated with lower odds of aggressive EOL care use than inpatient hospice care. Similarly, previous studies have reported that outpatient- or consultation-based palliative care is associated with lower rates of aggressive EOL care, including ICU admission, ED visits, and hospitalization (14, 39). These findings reflect the continuity and longitudinal follow-up inherent in consultation-based hospice care, which may facilitate earlier hospice involvement and ongoing supportive care (10). Home-based hospice care may reduce unnecessary acute healthcare utilization by allowing patients to receive care in familiar and comfortable environments (49).

This study had a few limitations. First, this study could not assess important clinical and preference-related factors such as performance status and patient preferences regarding EOL care (50), which may influence aggressive EOL care use. Second, because information on the timing of hospice referral was unavailable in our data, the analyzed interval might have included the waiting period before hospice initiation. Because aggressive care outcomes were ascertained over the same interval, a longer measured interval also corresponded to a longer period at risk for these events, which may have resulted in some overestimation of the observed associations. Accordingly, the findings should be interpreted as reflecting the association between the overall transition from the last SACT to hospice initiation and aggressive care rather than the effect of hospice referral timing. Third, because patients receiving hospice care in Korea generally forgo cardiopulmonary resuscitation and other life-sustaining treatments, ICU admission was expected to be uncommon. Nevertheless, this outcome is an internationally recognized indicator of aggressive EOL care and reflects important tendencies on this topic. Finally, diagnostic codes in the analytic dataset were available only at the 3-character ICD-10 level. Therefore, comorbidities included in the modified CCI were identified using broader 3-character ICD-10 categories based on previously developed ICD-10 comorbidity algorithms, rather than using more granular 4- or 5-character subcodes (33, 34). This may have limited the precision of comorbidity classification.

This study provides nationwide evidence that timely hospice initiation is associated with reduced aggressive EOL care in Korean patients with terminal cancer. Achieving timely hospice initiation may require earlier referral and consultation to hospice care in clinical practice, which may help reduce aggressive interventions near death (39). Despite the well-established benefits of early hospice initiation, structural and individual barriers continue to delay the timely transition to hospice care in clinical practice. To address these barriers, multifaceted strategies are needed, including clinician education and improved patient-provider communication to reframe hospice care as a quality-of-life-focused care (38). Further research examining healthcare utilization trajectories across this transition period and the factors that facilitate timely hospice initiation remains warranted.

## 5 Conclusions

A substantial proportion of patients with major cancers initiated hospice care >30 days after their last SACT, and the proportion with intervals >90 days increased over the study period. Longer intervals between the last SACT and hospice initiation were associated with progressively higher odds of ICU admissions, two or more ED visits, and composite outcomes before hospice care. These findings suggest that a timely transition to hospice care may be important to reduce aggressive healthcare utilization near the EOL in patients with terminal cancer.

## Data Availability

The datasets presented in this article are not readily available because the data supporting the findings of this study are obtainable from the K-cure (Korea-Clinical Data Utilization Network for Research Excellence). However, access to these data may be restricted due to their usage under approval policy for the current study, making them not publicly accessible. Nevertheless, interested parties can request access to the raw data from K-CURE, subject to the approval by the relevant review board upon reasonable inquiries. Requests to access the datasets should be directed to K-CURE (Korea-Clinical Data Utilization Network for Research Excellence), https://k-cure.mohw.go.kr.
