# Supplementary material for: Timing of hospice care initiation and aggressive care utilization in patients with cancer: a retrospective nationwide study in Korea
Source: Front Public Health. 2026 Aug 18;14:1909398. doi: 10.3389/fpubh.2026.1909398 (PMC13529667; doi:10.3389/fpubh.2026.1909398)
Supplement: Supplementary file 1 [file Table_1.docx]

| **Supplementary Table S1. Unadjusted association between timing of hospice initiation and aggressive care use** | | | | | | |
| --- | --- | --- | --- | --- | --- | --- |
| **Timing of hospice initiation** | **ICU admission, unadjusted OR (95% CI)** | **p-value** | **ED visits ≥2 times, unadjusted OR (95% CI)** | **p-value** | **ICU admission or ED visits≥2 times, unadjusted OR (95% CI)** | **p-value** |
| ≤30 days | Ref. |  | Ref. |  | Ref. |  |
| 31–60 days | 3.538 (2.870–4.361) | <0.0001 | 6.098(5.379-6.912) | <0.0001 | 5.453(4.885-6.086) | <0.0001 |
| 61–90 days | 5.695 (4.535–7.151) | <0.0001 | 14.025(12.299-15.994) | <0.0001 | 12.091(10.751-13.598) | <0.0001 |
| >90 days | 9.038 (7.577–10.781) | <0.0001 | 31.926(28.595-35.645) | <0.0001 | 25.646(23.296-28.234) | <0.0001 |

*ORs and 95% CIs were estimated using logistic regression models with hospice timing as the only independent variable.

| **Supplementary table S2. Supplementary Table 2. Operational definitions and reimbursement codes for hospice and cancer treatment** | | | |
| --- | --- | --- | --- |
| **Variable** | | **Operational definition** | **Reimbursement codes criteria** |
| **Hospice care use** | | Use of any Inpatient, Home-based, Consultation hospice care service | WA100–WA482, WB100–WB422, WC100–WC422, WD100–WD482, WE100–WE422, WF100–WF422, and IP111–IP450 series |
| **SACT** | **SACT drug use** | Prescription or administration of antineoplastic agents or drugs acting primarily against malignant neoplasms | Korean therapeutic classification codes 421 and 617, excluding appetite-stimulating agents |
|  | **SACT acting related codes** | Administration of anticancer agents through intravenous, intra-arterial, or subcutaneous injection, including infusion-pump use | KK151–KK156, KK158, and KK159 series |
| **Emergency department visit** | | Emergency medical care identified using emergency care reimbursement codes or a record from the emergency medicine department | VA100, VA200, VA210, VA220, VA300, VA310, VA320, VA400, VA500, VA510, VA520, VA800, VA810, and VA820 series; V11–V15, V18, V21–V23, and V26 series; AC101, AC103, and AC105 series; or emergency medicine department code 24 |
| **Intensive care unit admission** | | Admission to an intensive care unit identified using ICU reimbursement codes | AJ series |
